# The Relationship Between Breast Density and Breast Cancer Surgical Outcomes: A Systematic Review

**DOI:** 10.7759/cureus.57265

**Published:** 2024-03-30

**Authors:** Yousef Alalawi, Sultan Abdulrahman S Alamrani, Omar M Alruwaili, Ibrahim F Alzahrani, Abdullah M Al Madshush

**Affiliations:** 1 Department of Surgery, King Salman Armed Forces Hospital in the North-Western Region, Tabuk, SAU; 2 Medicine, University of Tabuk, Tabuk, SAU; 3 Medicine, Tabuk University, Faculty of Medicine, Tabuk, SAU

**Keywords:** breast surgery, breast carcinoma, surgical outcomes, breast cancer, breast density

## Abstract

This study aims to investigate the relationship between mammographic breast density and the surgical outcomes of breast cancer. PubMed, SCOPUS, Web of Science, Science Direct, and the Wiley Library were systematically searched for relevant literature. Rayyan QRCI was employed throughout this comprehensive process. Our results included ten studies with a total of 5017 women diagnosed with breast cancer. The follow-up duration ranged from 1 year to 15.1 years. Eight out of the twelve included studies reported that low mammographic breast density was significantly associated with no local recurrence, metachronous contralateral breast cancer, and fewer challenges in the preoperative and intraoperative phases. On the other hand, four studies reported that mammographic breast density is not linked to disease recurrence, survival, re-excision, or an incomplete clinical and pathological response. There is a significant association between low mammographic breast density and reduced challenges in the preoperative and intraoperative phases, as well as no local recurrence and fewer mastectomy cases. However, the link between mammographic breast density and disease recurrence, survival, re-excision, and incomplete clinical and pathological response is less clear, with some studies reporting no significant association. The findings suggest that mammographic breast density may play a role in certain aspects of breast cancer outcomes, but further research is needed to fully understand its impact.

## Introduction and background

Breast cancer continues to be the most often diagnosed cancer globally and the second leading cause of cancer-related death for women. The American Cancer Society predicts that, in 2024, approximately 310,720 new cases of invasive breast cancer will be diagnosed in women, along with about 56,500 new cases of ductal carcinoma in situ (DCIS). Tragically, an estimated 42,250 women are expected to lose their lives to breast cancer [1]. Screen film mammography has been mostly replaced with digital mammography in the last ten years, with mammography still being the modality utilized in ordinary clinical practice for screening and diagnosis. However, because mammography exposes patients to cumulative low-level X-ray radiation, it is not the best method for measuring breast density. A great deal of research has been done on alternative modalities, chief among these being digital breast tomosynthesis and magnetic resonance imaging [2,3].

Due to the different radiographic attenuation characteristics of fibroglandular tissue versus fat, mammographic density refers to the percentage of opaque (white) dense breast tissue compared to the radiolucent (dark) areas seen on mammography [4]. Mammographic density is typically expressed as a percent, which is computed by dividing the dense area by the total breast area [5].

Numerous studies have looked into the relationship between mammographic density and treatment outcomes as well as prognosis [6-8]. Different findings, meanwhile, have been produced by this research about the correlation between mammographic density characteristics and patient outcomes. Research indicates that there are ethnic differences in treatment response and breast cancer mortality [9,10], as well as a 20-30% variance in mammographic density that coincides with variation in breast cancer incidence [11]. It is unclear, though, if ethnic differences or the mammographic density phenotype that was utilized are confusing the link between mammographic density and the result. Crucially, nothing is known about how mammographic density modifications affect the nature and results of breast cancer treatment. Despite the aforementioned gaps, no evaluation of the literature has been done to determine the actual effects of mammographic breast density and mammographic density reduction after cancer treatment on the prognosis of women who have been diagnosed with or are receiving treatment for breast cancer. This highlights the necessity of conducting a thorough examination of published research to determine the connection between mammographic density and treatment results. The main objective of this comprehensive review is to investigate the relationship between breast density and the surgical outcomes of breast cancer.

## Review

The present systematic review adhered to the PRISMA (Preferred Reporting Items for Systematic Reviews and Meta-Analyses) standards [12].

Study design and duration

January 2024 marked the start of this systematic review.

Search strategy

Articles were determined through five main databases: PubMed, SCOPUS, Web of Science, Science Direct, and Wiley Library to comprise relevant data. We limited our search to English and considered each database's specific needs. The following keywords were transformed into PubMed Mesh terms or subject terms in Scopus and used to locate the pertinent studies: "Breast cancer," "Breast density," "Mammographic density," "Surgical outcomes," "Survival," "Recurrence," and "Re-excision." The Boolean operators "OR" "AND" and "NOT" matched the required keywords. Publications with full English text, available free articles, and human trials were among the search results.

Eligibility criteria

Inclusion Criteria

We considered articles that studied the relationship between mammographic breast density and the surgical outcomes of breast cancer and any study design discussing the required outcomes for inclusion in this review. Adults (>18 years), only human subjects, English language, and free, accessible articles were included.

Exclusion Criteria

In our evaluation approach, we excluded case reports, unpublished data, reviews, letters, conference abstracts, and insufficient data. After the investigators finished their eligibility review, any disagreements were discussed and resolved by the authors.

Data extraction

The outcomes of the search technique were verified twice with Rayyan QCRI [13]. The researchers added inclusion/exclusion criteria to the aggregated search results in order to evaluate the relevance of the titles and abstracts. The reviewers thoroughly read all of the papers that met the inclusion criteria. The authors talked about how to settle arguments. A previously developed data extraction form was used to upload the approved study. The authors extracted data about the study titles, authors, study year, country, participants, follow-up, menopausal status, type of surgery, patient outcome, and main outcomes. A separate sheet was created for the risk of bias assessment.

Strategy for data synthesis

The summary tables created utilizing information from relevant studies provide a qualitative assessment of the research findings and their constituent parts. The best technique for making use of the data from the included study articles was chosen after the data for the systematic review was gathered.

Risk of bias assessment

The Joanna Briggs Institute's (JBI) [14] key assessment criteria for studies providing prevalence data were applied in order to evaluate the research's quality. This technique was used to evaluate studies using nine questions. The question was given a score of 1 if the answer was in the affirmative. Any response that was no, unclear, or not applicable received a score of 0. For overall quality, ratings of less than 4, 5 to 7, and more than 8 were regarded as low, moderate, and high quality, respectively. The scholars assessed the caliber of the research they carried out, and disagreements were settled by discussion.

Results

**Figure 1:**
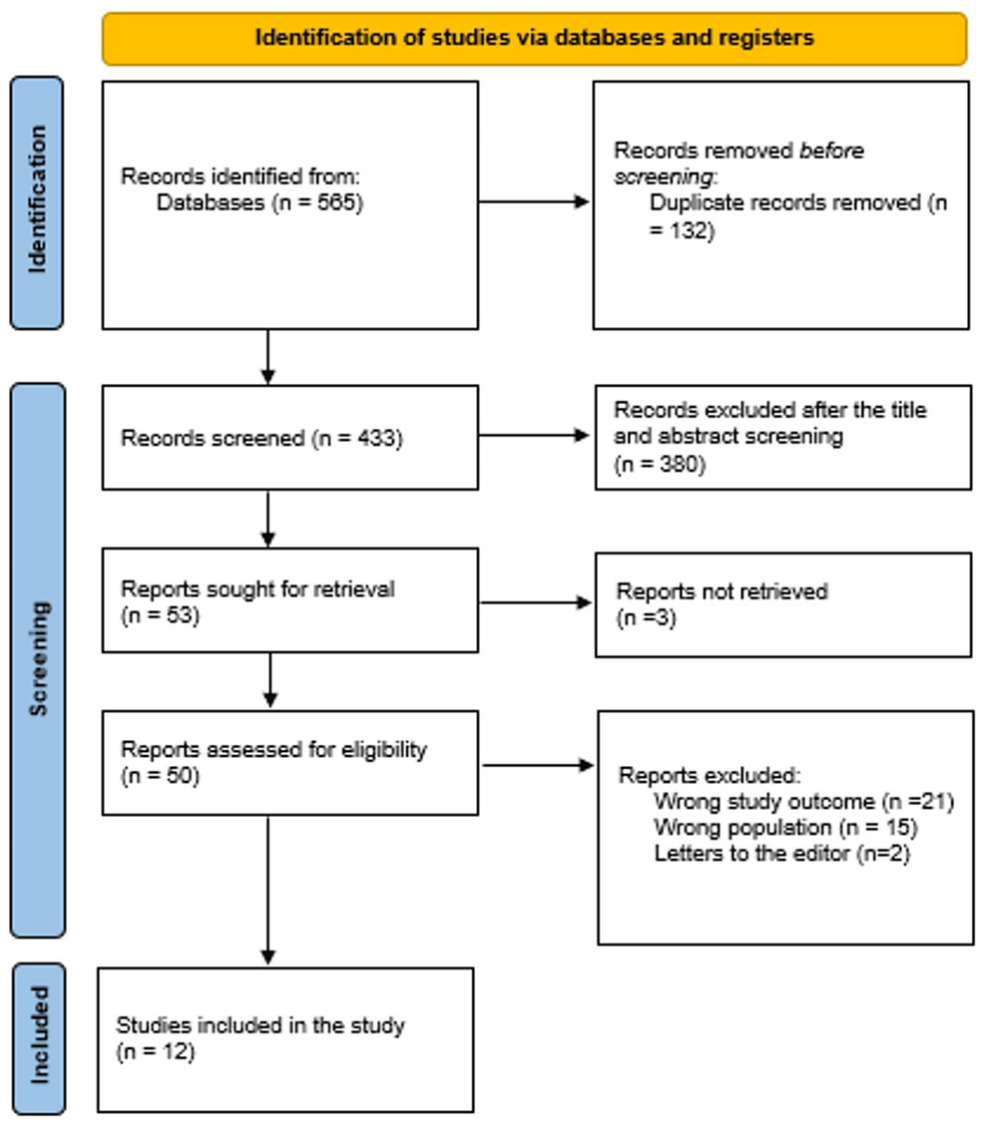
PRISMA flowchart summarizes the study selection process. PRISMA: Preferred Reporting Items for Systematic Reviews and Meta-Analyses.

The systematic search yielded a total of 565 study articles, of which we deleted 132 duplicates. Title and abstract screening were conducted on 433 studies, and 380 were excluded. We retrieved 53 reports and excluded only 3 articles. Finally, 50 studies were screened for full-text assessment; 21 were excluded for the wrong study outcomes, 15 for the wrong population type, and 2 articles were letters to the editors. Twelve eligible study articles were included in this systematic review. A summary of the study selection process is presented in Figure 1. 

Table 1 presents the sociodemographic characteristics of the included study articles [15-26]. Our results included 12 studies, with 5017 women diagnosed with breast cancer. Nine studies were case-control studies [15-18,21-23,25,26], two were retrospective in nature [20,24], and one was prospective in nature.

**Table 1:** Sociodemographic characteristics of the included participants. NM: not-mentioned, PD: percentage density.

Study	Study design	Country	Participants	Mean age
Eriksson et al. [15]	Case-control	Sweden	PD <25% (n = 1,329) PD ≥25% (n = 445)	50-74
Cil et al. [16]	Case-control	Canada	Low density (99), intermediate density (107), high density (129)	63.5
Faermann et al. [17]	Case-control	Canada	165 dense and 105 not dense	50.2
Edwards et al. [18]	Case-control	USA	Fatty (n = 80, 20.1 %), scattered (n = 170, 42.7 %), heterogeneous (n = 121, 30.4 %), and extreme (n = 27, 6.7%)	42-71
Bani et al. [19]	Prospective cohort	Germany	565	55.8 ± 11.6
Zdanowski et al. [20]	Retrospective cohort	Sweden	302	41-68
Park et al. [21]	Case-control	USA	136	NM
Huang et al. [22]	Case-control	Taiwan	121 cases and 121 control	51.4
Sadaka et al. [23]	Case-control	Egypt	23 with low breast density and 64 high breast density	NM
Kim et al. [24]	Retrospective cohort	Korea	Recurrence group (12) and nonrecurrence (68)	44.4 ± 6.5
Walsh et al. [25]	Case-control	USA	Minimum density 1/2 (n = 487) 3/4 (n = 214	28-90
Agarwal et al. [26]	Case-control	Australia	127	28-97

Table 2 presents the clinical characteristics. The follow-up duration ranged from 1 year [18] to 15.1 years [16]. Eight out of the twelve included studies reported that low mammographic breast density was significantly associated with no local recurrence, metachronous contralateral breast cancer, and fewer challenges in the preoperative and intraoperative phases [16,17,19-24]. On the other hand, four studies reported that mammographic breast density is not linked to disease recurrence, survival, re-excision, or an incomplete clinical and pathological response [15,18,25,26].

**Table 2:** Clinical characteristics and outcomes of the included studies. NM: not mentioned, JBI: the Joanna Briggs Institute for risk of bias assessment.

Study	Follow-up duration (years)	Menopausal status	Type of surgery	Patient outcome	Main outcomes	JBI
Eriksson et al. [15]	10	Post-menopausal	Breast conservative surgery and mastectomy	Local recurrence and locoregional recurrence	Elevated mammography density is a risk factor for local and locoregional recurrence, not related to distant metastases or survival.	Moderate
Cil et al. [16]	0.9–15.1	The majority 219 (65.4%) are postmenopausal	Breast conservative surgery	Local recurrence	The study found no local disease recurrence among 34 women with poor breast density who did not undergo radiation, indicating that their decision to forgo radiation was not impaired.	Moderate
Faermann et al. [17]	6	NM	Mastectomy	local recurrence, re-excision, involved margins, metachronous contralateral breast cancer, late distant recurrence, and death	Preoperative breast magnetic resonance imaging in dense breast patients may improve long-term and surgical outcomes, with lower incidence of local recurrence and metachronous contralateral breast cancer, and fewer re-excision operations, though the difference is not statistically significant.	High
Edwards et al. [18]	1	NM	Breast conservative surgery	Re-excision	The study found no significant impact of mammographic density on margin involvement, conversion to mastectomy, or extra margin removal during breast conservative surgery, suggesting that mammographic density should not influence surgical decisions.	Moderate
Bani et al. [19]	NM	NM	Breast conservative surgery	Recurrence	Mammographic density is a key risk indicator in breast-conserving therapy, as higher density increases challenges in tumor localization and removal.	Moderate
Zdanowski et al. [20]	3.4	Majority were postmenopausal	The type of surgery was not detected	Recurrence and survival	A study found that patients with very dense breasts had lower recurrence-free and breast cancer-specific survival rates compared to those with less dense breasts.	Moderate
Park et al. [21]	7.7	NM	Breast conservative surgery	Local recurrence	High mammographic breast density predicts local recurrence after breast conservative surgery and radiotherapy, but not distant recurrence. Obesity and high mammographic breast density have a negative confounding.	High
Huang et al. [22]	7	Majority were >50 years	Modified radical mastectomy	Local recurrence	For invasive breast cancer, high mammographic breast density (>50% density) is a strong independent predictor of local recurrence following Modified radical mastectomy.	High
Sadaka et al. [23]		More than half (55.2%) were above 50 years	Modified radical mastectomy	Local recurrence	The local recurrence of invasive breast cancer in female patients is significantly influenced by mammographic breast density.	Moderate
Kim et al. [24]	3.1-5	15 (18.75%) were postmenopausal	Breast conservative surgery and received adjuvant tamoxifen treatment	Recurrence	Breast cancer patients receiving adjuvant tamoxifen showed changes in breast density as an independent risk factor for cancer recurrence, as determined by 3D magnetic resonance imaging before and after treatment.	Moderate
Walsh et al. [25]	0–5.3	NM	Breast conservative surgery	Re-excision	The study found that while women with thick breasts are more likely to need surgery, their breast density did not negatively affect their disease-free survival.	Moderate
Agarwal et al. [26]	9.3	NM	Breast conservative surgery	Clinical and pathological response	High mammographic breast density is not linked to pathologic complete response or breast cancer death, but rather to a lesser clinical complete response and a higher rate of relapse.	Moderate

Discussion

The available research indicates a dearth of information about surgical outcomes in particular and contradictory findings when using mammographic density to evaluate the course of breast cancer treatment. A number of factors, such as mammographic breast density assessment techniques, study design, sample selection, mammographic breast density measurement time, surgical type, varying follow-up lengths, and confounder adjustment, could account for the contradictory data. To use data collected from radiological imaging for breast cancer prediction and treatment efficacy monitoring, a deeper comprehension of these parameters and their implications is necessary.

This review demonstrated that low mammographic breast density was significantly associated with no local recurrence, no metachronous contralateral breast cancer, higher survival rates, and fewer challenges in the preoperative and intraoperative phases [16,17,19-24]. Similarly, Kanbayti et al., in their systematic review and meta-analysis, reported that there is limited evidence to suggest that having a high baseline mammographic breast density raises the risk of breast cancer mortality, complete blood count, and recurrence. On the other hand, mammographic breast density reduction is linked to a lower incidence of complete blood count, breast cancer-related mortality, and breast cancer recurrence. These results present a promising picture of the effectiveness of mammographic breast density for evaluating breast cancer treatment outcomes or enhancing the functionality of the available models for predicting breast cancer treatment outcomes [27].

The ability of dense breasts to limit drug delivery to the tumor site [28], the existence of residual tumor volumes in extremely dense breasts compared to fatty breasts [18], and the increased risk of cancer in dense breast women [22] are some of the theories put forth to explain the low recurrence in dense breasts.

Crucially, in order to use mammographic density reduction as a stand-in marker for treatment outcomes, we need to pinpoint the metrics or phenotypes that most accurately reflect changes in mammographic breast density, particularly alterations in the fibroglandular tissue composition, which is the primary tissue that affects outcomes. Mammographic density reduction has been linked to better results in other phenotypes, but there are not nearly enough data to make firm judgments. Three investigations have demonstrated that, in women for whom area-based approaches were unable to detect changes in mammographic breast density following endocrine therapy, volumetric assessments from mammograms and magnetic resonance imaging could [29-31]. However, this research did not examine the patient outcome from such a reduction in mammographic breast density. Consequently, more research is required to evaluate the connection between variations in volumetric density and patient outcomes. Considering that volumetric instruments need raw data, which can be challenging to get, automated mammographic breast density measurement techniques that take area and depth data into consideration ought to be investigated. These methods could provide a more accurate evaluation of mammographic breast density reduction as a factor influencing patient outcomes. Results may vary depending on therapy interventions, length of treatment, and treatment regimes. The majority of the examined studies have limitations because they failed to take these parameters' impact on the relationship between mammographic breast density and treatment results into consideration. Furthermore, it was challenging to determine if mammographic breast density is an independent or intermediate marker of treatment outcome because nearly all of the research examined relationships with minimal focus on outcome prediction.

On the other hand, four studies reported that mammographic breast density is not linked to disease recurrence, survival, re-excision, or an incomplete clinical and pathological response [15,18,25,26]. Tracking changes in density is complicated by the short follow-up intervals in published studies. Based on the observation that individuals with low mammographic breast density saw slight reductions that could not have been enough to affect mortality, it seems that mammographic breast density is another crucial factor to take into account [32,33]. Further research should be done on the possibility that racial characteristics contribute to the negative correlation between mammographic density reduction and recurrence [24].

## Conclusions

Low mammographic breast density is significantly associated with reduced challenges in the preoperative and intraoperative phases, no local recurrence, and fewer mastectomy cases. However, the link between mammographic breast density and disease recurrence, survival, re-excision, and incomplete clinical and pathological response is less clear, with some studies reporting no significant association. The findings suggest that mammographic breast density may play a role in certain aspects of breast cancer outcomes, but further research is needed to fully understand its impact.
